# The mechanism of macroautophagy: The movie

**DOI:** 10.1080/27694127.2022.2096115

**Published:** 2022-09-14

**Authors:** Fulvio Reggiori, Patricia Boya, David da Costa, Zvulun Elazar, Eeva-Liisa Eskelinen, Judith Farrés, Sebastian Guettler, Claudine Kraft, Heinz Jungbluth, Ana Martinez, Etienne Morel, Ole Pless, Tassula Proikas-Cezanne, Anne Simonsen

**Affiliations:** a Department of Biomedical Sciences of Cells & Systems, University of Groningen, University Medical Centre Groningen, 9713, AV Groningen, The Netherlands; b Department of Biomedicine, Aarhus University, Ole Worms Allé 4, 8000 Aarhus C, Denmark; c Aarhus Institute of Advanced Studies (AIAS), Aarhus University, Høegh-Guldbergs Gade 6B, 8000 Aarhus C, Denmark; d Centro de Investigaciones Biológicas Margarita Salas (CSIC), Ramiro de Maeztu 9, 28040 Madrid, Spain; e Adjuvatis, 60 avenue Rockfeller, 69008 Lyon, France; f Department of Bimolecular Sciences, The Weizmann Institute of Science, 76100, Rehovot, Israel; g Institute of Biomedicine, University of Turku, 20520 Turku, Finland; h Anaxomics Biotech S.L., c. Diputació, 237 1-1 08007 Barcelona, Spain; i Cancer Research UK Cancer Therapeutics Unit, The Institute of Cancer Research, London SM2 5NG, United Kingdom; j Institute of Biochemistry and Molecular Biology, ZBMZ, Faculty of Medicine, University of Freiburg, 79104 Freiburg, Germany; k CIBSS - Centre for Integrative Biological Signalling Studies, University of Freiburg, 79104 Freiburg, Germany; l Randall Division of Cell and Molecular Biophysics, Muscle Signalling Section, King’s College London, London, United Kingdom; m Department of Paediatric Neurology, Evelina Children’s Hospital, Guy’s & St Thomas’ NHS Foundation Trust, London, United Kingdom; n Centro de Investigaciones Biomedicas en Red en Enfermedades Neurodegenerativas (CIBERNED), Instituto de Salud Carlos III, 28029 Madrid, Spain; o Université Paris Cité, INSERM UMR-S1151, CNRS UMR-S8253, Institut Necker Enfants Malades, F-75015 Paris, France; p Fraunhofer Institute for Translational Medicine and Pharmacology ITMP, ScreeningPort, D-22525 Hamburg, Germany; q Interfaculty Institute of Cell Biology, Eberhard Karls University Tübingen, Auf der Morgenstelle 15, D-72076 Tübingen, Germany; r Department of Molecular Medicine, Institute of Basic Medical Sciences and Centre for Cancer Cell Reprogramming, Institute of Clinical Medicine, Faculty of Medicine, University of Oslo, 1112 Blindern, 0317 Oslo, Norway

## Abstract

This animated movie presents the mechanism of macroautophagy, hereafter autophagy, by showing the molecular features of the formation of autophagosomes, the hallmark organelle of this intracellular catabolic pathway. It is based on our current knowledge and it also illustrates how autophagosomes can recognize and eliminate selected cargoes.

Macroautophagy, hereafter autophagy, is a highly conserved intracellular lysosomal degradation process, which is essential to maintain cellular homeostasis by turning over unwanted cytoplasmic structures and recycling their basic components [1]. This catabolic pathway targets excess or dysfunctional proteins, protein complexes and organelles, but also invading pathogens, by sequestering them within double-membrane vesicles called autophagosomes, and delivering them to lysosomes/vacuoles for degradation [2–4]. The interest in autophagy has grown exponentially and attracted the attention of basic and translational researchers, the pharmaceutical industry, but also the public, due to a series of seminal discoveries revealing its involvement in numerous physiological processes and pathological conditions. Autophagy participates in, e.g., the adaptation to starvation and other stresses, development and cell differentiation, and immunity and lifespan extension [5,6]. Moreover, autophagy plays a relevant role in the pathophysiology of neurodegenerative, cardiovascular, chronic inflammatory, muscular and autoimmune diseases, and some malignancies as well [5–8]. More recently, defective autophagy has been implicated in a rapidly expanding group of early onset Mendelian neurodevelopmental and neurological disorders with variable multisystem involvement, the “congenital disorders of autophagy” [9], emphasizing the importance of normally functioning autophagy for neuronal development and maintenance throughout life [10]. Crucially, it has been shown that autophagy modulation is a potentially effective therapy to prevent or cure diseases, including specific types of tumors, muscular dystrophies and neurodegenerative disorders [5,11–13].

*Driving next-generation autophagy researchers towards translation* (DRIVE) is a pan-European Autophagy researchers’ consortium [14]. This Marie Skłodowska-Curie Early Training Network was approved under the European Union’s Horizon 2020 Research and Innovation Program and has been funded over a period of 5 years. Within DRIVE, 14 European research teams from academia and industry have trained 15 PhD students through basic, applied, cross-disciplinary and collaborative autophagy research [14]. One of the goals of DRIVE was to create a movie to introduce the mechanistic principles of autophagy to colleagues, students and the general public. Enormous advances in our molecular understanding of autophagy have been made in the past two decades and a visual representation of this cellular pathway makes it possible to easily explain to non-specialists how the field currently thinks about the process of autophagy and how it is currently thought to work. In particular, the movie illustrates how autophagosomes could be generated de novo and how they can specifically recognize and turn over selected cargoes. With this movie, which can be find at https://www.youtube.com/watch?v=Gc9gx33GvF0, we hope to provide a simple but clear look into autophagy, hopefully conveying our excitement in investigating this unique cellular process that could lead to important medical applications.

Good vision!
